# A Wavelet-Based Noise Reduction Algorithm and Its Clinical Evaluation in Cochlear Implants

**DOI:** 10.1371/journal.pone.0075662

**Published:** 2013-09-26

**Authors:** Hua Ye, Guang Deng, Stefan J. Mauger, Adam A. Hersbach, Pam W. Dawson, John M. Heasman

**Affiliations:** 1 Department of Electronic Engineering, La Trobe University, Melbourne, Victoria, Australia; 2 Cochlear Limited, Melbourne, Victoria, Australia; 3 HEARing CRC, Melbourne, Victoria, Australia; University of Adelaide, Australia

## Abstract

Noise reduction is often essential for cochlear implant (CI) recipients to achieve acceptable speech perception in noisy environments. Most noise reduction algorithms applied to audio signals are based on time-frequency representations of the input, such as the Fourier transform. Algorithms based on other representations may also be able to provide comparable or improved speech perception and listening quality improvements. In this paper, a noise reduction algorithm for CI sound processing is proposed based on the wavelet transform. The algorithm uses a dual-tree complex discrete wavelet transform followed by shrinkage of the wavelet coefficients based on a statistical estimation of the variance of the noise. The proposed noise reduction algorithm was evaluated by comparing its performance to those of many existing wavelet-based algorithms. The speech transmission index (STI) of the proposed algorithm is significantly better than other tested algorithms for the speech-weighted noise of different levels of signal to noise ratio. The effectiveness of the proposed system was clinically evaluated with CI recipients. A significant improvement in speech perception of 1.9 dB was found on average in speech weighted noise.

## Introduction

In an ideal, quiet listening environment, modern cochlear implant (CI) devices are capable of restoring speech perception to most recipients [1]. In a more realistic, noisy listening environment, however, the level of speech perception degrades rapidly with the increased noise level [2]. Studies evaluating single channel noise reduction algorithms for CIs have reported significant speech perception improvements of 24 percentage points in babble noise [2], 2.1 dB speech reception thresholds [3] and 19 percentage points [4] in speech weighted noise. This work is inspired by the improvement of speech understanding due to noise reduction techniques. We aim to explore different ways to further improve speech understanding in noisy listening environments. In the following, we briefly review noise reduction techniques related to our work.

Early multichannel stimulation strategies used feature extraction methods to present formant information to the implant electrode array [5]. A noise reduction method was shown to provide speech understanding benefit using such a feature extraction based stimulation strategy [6]. The current commercially available stimulation strategies (continuous interleaved sampling (CIS) and advanced combination encoding (ACE™) [7]) use time-frequency decompositions of the signal to obtain a number of band-pass channels. The energy in each channel is then calculated and used to determine the stimulus level for each electrode [7]. These stimulation strategies superseded feature based stimulation strategies due to improved speech understanding outcomes. Using ACE™ and CIS stimulation strategies, two acute laboratory studies tested the benefit of noise reduction by using hearing aids algorithms to pre-process signals before being presented to the cochlear processor, finding improvements in some noise types [8], [9]. Implementation in take home behind-the-ear (BTE) devices for daily use has also shown similar improvements [10], [11]. Directional microphones, be this software or hardware in implementation have also shown improvements of speech understanding in noise [11], [12].

Traditional approaches to single-channel noise reduction in CI devices, as well as general speech enhancement for normal listeners, have been spectral subtraction or other spectral modification applied to the Fourier transform coefficients [13], [14], [15], [16]. While the Fourier transform is well suited to capture stationary features of signals, the highly localized wavelet transform is better suited for non-stationary signals such as speech [17]. Wavelet transforms have been used in many speech enhancement applications. For example, Lei and Tung [18] proposed a system using the wavelet packet transform to emulate the subband decomposition known to occur in human hearing. This implementation is also thought to reduce the effect of ‘musical noise’. The wavelet transform was also proposed as an alternative to the Fourier transform for signal decomposition in CI devices [17], [19], [20], [21].

Although wavelets have been used to good effect in other areas of noise reduction and have been discussed as an alternative to Fourier decomposition for CIs, there has been no report on using wavelet transforms specifically for noise reduction in CI devices. The objective of this study is to investigate the clinical application of the wavelet transform as a tool in noise reduction for CI recipients. In particular, a dual-tree complex wavelet transform based noise reduction technique will be proposed and clinically evaluated with CI recipients.

## Wavelet-based Noise Reduction Algorithm

### The Overall System Structure

**Figure 1:**
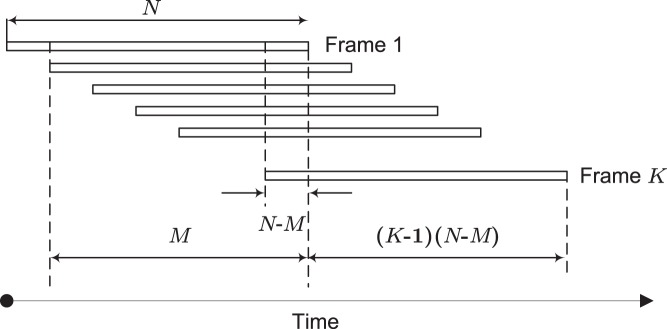
Overlapping frame arrangement. Each frame has *N* samples with *M* samples overlapped with the previous frame. The number of frames used for averaging is *K*. After the processing of a new frame, 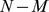 processed samples will be output.

The proposed algorithm consists of the following major steps:

- The signal 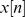 is buffered as overlapping frames. There are two parameters: length of each frame, denoted by *N* and number of signal samples overlapped with the previous frame, denoted by *M*. The settings of these two parameters are based on a trade-off between latency, computational complexity and performance of denoising. This frame arrangement is shown in **Figure 1**.
- Each frame is processed by the wavelet-based denoising algorithm.
- It can be seen from **Figure 1** that there will be multiple (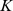) processed values for each sample - each coming from a different frame. Assuming that the residual noise in different processed frames are not correlated to each other, averaging these values (indicated between two dash lines in **Figure 1**) helps further reduce the residual noise.

**Figure 2:**
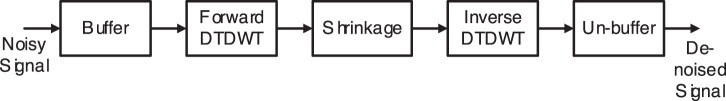
Block diagram of the dual-tree complex discrete wavelet transform (DTDWT) based noise reduction system.

A block diagram of the overall noise reduction system is shown in **Figure 2**. The system works as a pre-processor such that its output is fed to the input of the CI device. Therefore, proper arrangement must be made to ensure the continuous flow of output data while allowing enough data samples in each frame for accurate calculation of the needed statistics. The parameters 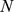, 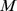 and 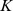 were selected based on experiments with a set of recorded voice samples corrupted with noise. The parameters were set at 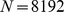, 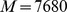, 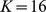 and 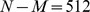 for a sampling rate of 16 kHz.

### Dual-tree Complex Wavelet Transform

The discrete wavelet transform (DWT) comes in several forms. The critically-sampled (standard) form of the transform provides the most compact representation. However, it is shift-variant, which is undesirable for speech enhancement. To overcome the shift-variance problem, noise reduction based on shift-invariant wavelet transforms [22] have been extensively studied. One of such transforms is the stationary (or undecimated) DWT [23]. However, a major drawback of the stationary DWT (SWT) is its computational cost.

Another one is the dual-tree complex discrete wavelet transform [24]. It consists of two specifically designed critically-sampled DWTs in parallel applied to the same input data. The subband signals of these two DWTs can be interpreted as the real and imaginary parts of a complex wavelet transform that is nearly shift-invariant. Since the transform equals to two standard DWTs, the dual-tree complex wavelet transform is more attractive than the SWT in terms of computational complexity. In this work, we adopt Sendur and Selesnick’s [24] implementation of the dual-tree complex wavelet transform (DTDWT).

### The Wavelet Shrinkage and Thresholding

A typical wavelet-based noise reduction algorithm involves shrinkage and thresholding of wavelet coefficients [22]. Using shrinkage with DTDWT, Sendur and Selesnick [24] have achieved better image denoising results than with critically-sampled wavelet transforms. The proposed algorithm is inspired by their work. For each frame, the proposed algorithm has the following major steps:

- Let 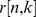 and 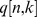 represent the 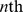 wavelet coefficients of the two trees at the 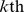 level. Let 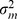 be the estimated variance by using the median absolute deviation (MAD) estimator [22], where *m* is the frame index. This estimate is further smoothed by using
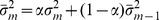
(1)where 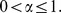 In our experiment, best estimation was given when 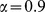. This was determined using a bench simulation of a set of data to maximize the speech transmission index (STI) (more details are given in subsection “Simulation experiments”).
- Treating the two trees as a complex signal 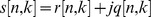, where 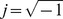, we want to denoise its amplitude: 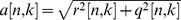. Due to the complexity of the statistical modelling of the amplitude, the maximum a posteriori probability (MAP) estimation algorithm is very computationally intensive. This is not suitable for real-time processing in the cochlear implant device. To solve this problem, we borrow the idea of shrinkage/thresholding function which is obtained by solving the following MAP estimation problem. We assume the additive signal model
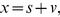
(2)where *s *is the true signal to be estimated and *v *is the noise. To simplify the algorithmic development and the algorithm, we assume that the noise *v* follows an independent and identically distributed (i.i.d) zero mean Gaussian distribution 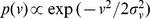 and the prior for the true amplitude *s* follows another zero mean i.i.d Gaussian 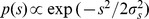. Solving the following MAP problem
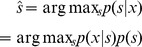
we obtain
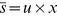
(3)where
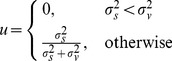
(4)is called the shrinkage/thresholding function.

- In this work we modify the above shrinkage/thresholding function to process the amplitude signal as follows
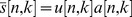
(5)where 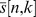 is the denoised amplitude and

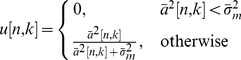
(6)In the above equation, 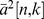 is an estimate of the signal energy and is the result of applying a low pass filter on the square of the amplitude signal 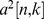. In this work, we use a simple 7-tap FIR filter with equal filter coefficient of 1/7. From a signal processing point of view, the variance represents the energy of the signal. Therefore, we use the signal energy in (6) to replace the signal variance in (4). In addition, the denoising of the amplitude of the signal is equivalent to denoising of the two trees using the same function 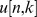 as follows
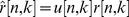
(7)and



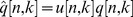
(8)This process is repeated for all levels of wavelet coefficients. The inverse dual-tree transform produces the denoised signal.

### Performance Evaluation and Clinical Validation in Cochlear Implants

#### Simulation experiments

Computer simulations of the proposed DTDWT algorithm and a range of well-established wavelet algorithms were performed to compare and predict the algorithms performance. The simulation experiments also provided the selection of the parameters of the proposed algorithm which were later applied in the clinical test.

Experiments were performed on two sets of recorded speech data. One data set was the Bamford-Kowal-Bench (BKB)-like sentences developed by the Cooperative Research Centre for Cochlear Implant and Hearing Aid Innovation [25]. The sentences were corrupted with speech weighted noise (SWN) at a signal-to-noise ratio (SNR) of 0 dB. The various parameters of the proposed algorithms were also trained on this set of data. The other data set was the NOIZEUS corpus developed at the University of Texas at Dallas [26]. These sentences are corrupted by 8 types of noise: train, babble, car, exhibition hall, restaurant, street, airport and train-station.

In the measurement of denoising performance, the speech transmission index (STI) was used to indicate how close the denoised signal was to the original clean target signal. This method has been shown to provide strong correlations with both modulated and non-modulated noise types [27]. Speech without noise added results in an STI of 1, as the signal becomes noisier the STI decreases, until the speech is totally dominated by noise will result in an STI near 0. The normalized correlation speech based STI metric proposed by Goldsworthy and Greenberg [28] was adopted in the calculation, because of its suitability in predicting speech intelligibility for CI users when non-linear signal processing is used. This is confirmed in a further study of speech intelligibility in cochlear implant simulations [29].

**Figure 3:**
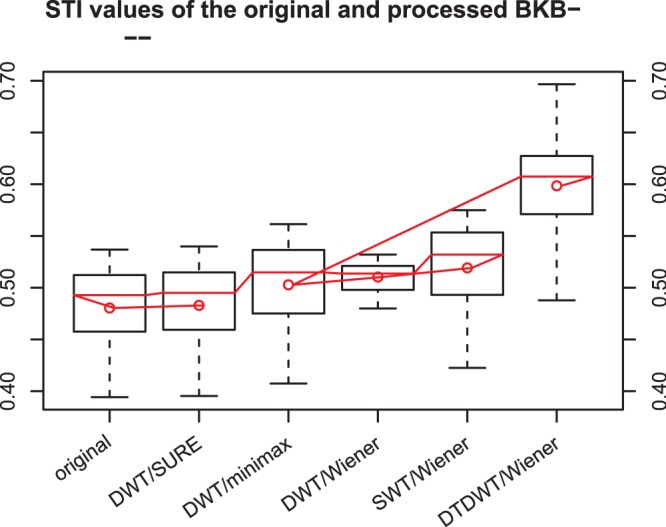
The box-plots of the STI values of the original and denoised BKB-like data. The speech signals were corrupted with speech weighted noise (SWN) at an SNR of 0 dB. The red dots indicate the mean values in the corresponding cases. The wavelets employed in DWT and SWT were the Daubechies-20 wavelets. The number of levels in decomposition was fixed at 6 for all cases.

**Figure 4:**
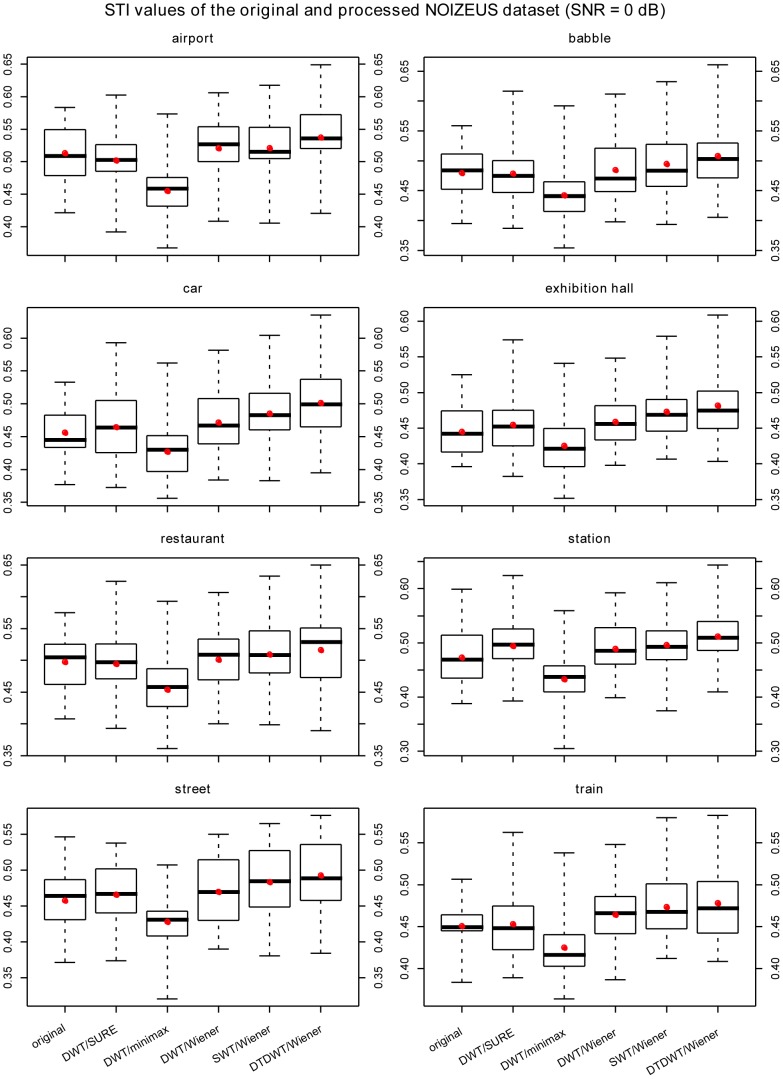
The box-plots of the STI values for NOIZEUS data at SNR = 0 dB. The speech signals were corrupted with 8 types of noise. The red dots indicate the mean values in the corresponding cases. The wavelets employed in DWT and SWT were the Daubechies-20 wavelets. The number of levels in decomposition was fixed at 6 for all cases.

**Figure 5:**
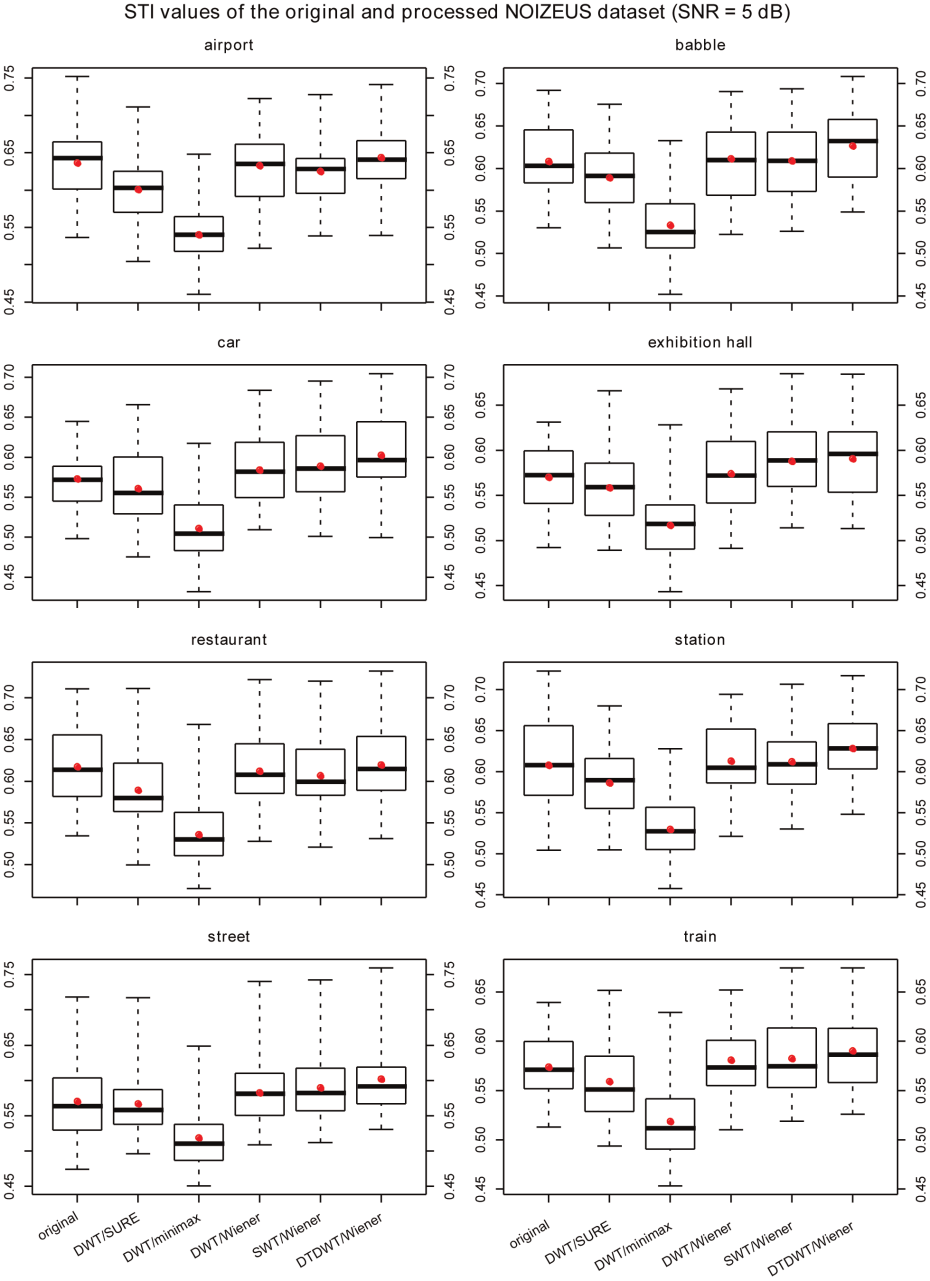
The box-plots of the STI values for NOIZEUS data at SNR = 5 dB. The speech signals were corrupted with 8 types of noise. The red dots indicate the mean values in the corresponding cases. The wavelets employed in DWT and SWT were the Daubechies-20 wavelets. The number of levels in decomposition was fixed at 6 for all cases.

**Figure 6:**
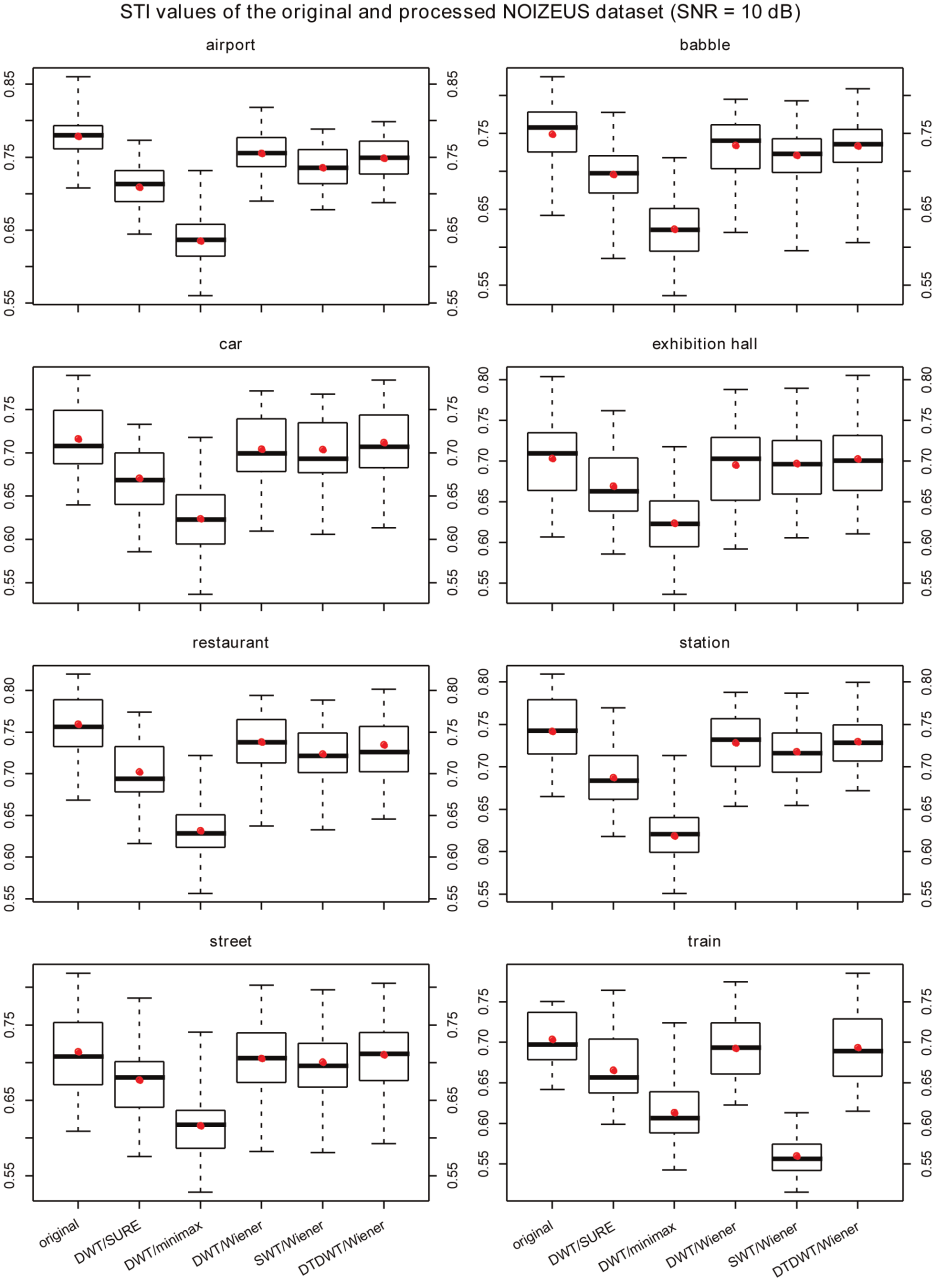
The box-plots of the STI values for NOIZEUS data at SNR = 10 dB. The speech signals were corrupted with 8 types of noise. The red dots indicate the mean values in the corresponding cases. The wavelets employed in DWT and SWT were the Daubechies-20 wavelets. The number of levels in decomposition was fixed at 6 for all cases.

The results are summarized in **Figure 3**, 4, 5, **6** where box-plots of the STI values for the BKB-like data corrupted with SWN at the SNR value of 0 dB (**Figure 3**), NOIZEUS data for the 8 noise conditions at the SNR values of 0 dB, 5 dB and 10 dB (**Figures 4** **,** **5** **,** **6**) were given. To compare the proposed algorithm to some conventional wavelet-based noise reduction algorithms, the results for several different forms of wavelets and different thresholding strategies are also listed in the figures. The forms of wavelets included DWT, SWT and DTDWT. The thresholding strategies were Stein’s Unbiased Risk Estimate (SURE), minimax method [22] and Wiener filtering. The results clearly show that the proposed algorithm consistently performs better than the other conventional wavelet-based noise reduction algorithms. Looking more closely at **Figures 4**, 5, and **6**, one may see that the advantage of the proposed algorithm over others is not very obvious in several noise conditions such as exhibition hall, restaurant and train. However, in most of those conditions, the proposed algorithm still outperforms the other algorithms in terms of the average STI value. It should be noted that at SNR values at or greater than 10 dB, all the noise reduction algorithms produced STI values that were lower than the unprocessed ones, although the proposed algorithm still outperformed other algorithms for most noise types. This suggests that the noise reduction algorithms should be turned off in relatively high SNR environments. For this reason, we have not included the results for SNR values greater than 10 dB.

#### Clinical test design and results

Nine adults implanted with a Cochlear™ Nucleus® CI system participated in the study.

This study was approved by the Royal Victorian Eye and Ear Hospital Human Research Ethics Committee (HREC 07/754H). All participants in this study gave written informed consent as approved by the Royal Victorian Eye and Ear Hospital Human Research Ethics Committee. All participants were current users of the Freedom™ sound processor. The ages of subjects at the time of testing range from 41 to 82 years and the duration of implant use range from 1.1 to 8.9 years. Total stimulation rate ranged from 4000 to 9600 pulses per second (pps). The biographical data of all subjects is summarized in **Table 1**.

**Table 1:** The biographical information of the clinical test subjects.

Subject	Age	Years ofimplant use	Stimulus rate(per electrode)	Maxima
1	79	3.9	720	12
2	62	1.6	900	8
3	68	3.3	1200	8
4	78	1.1	900	8
5	42	2.1	900	8
6	70	1.1	1200	8
7	60	6.8	900	8
8	67	8.9	500	8
9	82	7.2	1200	8

The clinical test used a repeated measure, single-subject design in which each subject served as their own control. The advanced combination encoder (ACE™) with and without DTDWT pre-processing were compared for each subject with sentences presented in SWN or 20-talker babble noise.

An adaptive speech reception threshold (SRT) test using BKB-like sentences provided the SNR for 50% morpheme intelligibility [11], [28]. BKB-like sentences were developed with region independent Australian vocabulary familiar to 5 year old children containing between four to six words. Sentences were recorded from a female adult Australian speaker, and noise was either speech-weighted noise, or 20-talker babble noise made from both female and male talkers. An adaptive test used in previous noise reduction studies [3], [11] has the advantage of having no floor or ceiling effects as can be found with fixed level testing due to the wide CI performance range. Morphemic scoring was chosen since it increases the number of items on which a sentence is scored and random measurement error in a score is predicted to decrease with an increase in the number of items responsible for that score [30], [31]. Two lists (32 sentences) were used to calculate a single SRT. The sentences were presented through a loudspeaker located 1.2 m directly in front of the subject. The speech was presented at 65 dB SPL (RMS), whilst the competing noise was adapted according to the accuracy of the subjects’ responses. The competing noise was decreased in level if the subject scored <50% morphemes correct and increased if the subject scored ≥50% morphemes correct. It was presented at the new, adapted presentation level for 3 seconds prior to the next target sentence. Prior to the initial sentence, it was presented for 12 seconds and thereafter presented continuously between sentences. The adaptive rule used in the Hearing in Noise Test (HINT) test was employed, with a step size of 4 dB for the first 4 sentences and a 2 dB step size for the remaining sentences [32]. The starting SNR was 5 dB. The SRT was calculated according to the HINT rule and was the average of the SNRs for sentences 5 to 32, in addition to the SNR at which sentence 33 would have been presented on the basis of the subject’s response to sentence 32.

Each subject listened to the sound using their Freedom processor, each programmed with ADRO®+Autosensitivity™ [1]. For the ACE condition, the sentences were presented unprocessed. For the implementation of the DTDWT program, the noisy speech signal was pre-processed by the DTDWT algorithm running on a computer-based real-time system and the denoised signal was presented to the subject. The order in which the ACE and DTDWT programs were tested was counterbalanced across the group. Data for analysis was the mean of two SRTs for each of the ACE and DTDWT programs.

**Figure 7:**
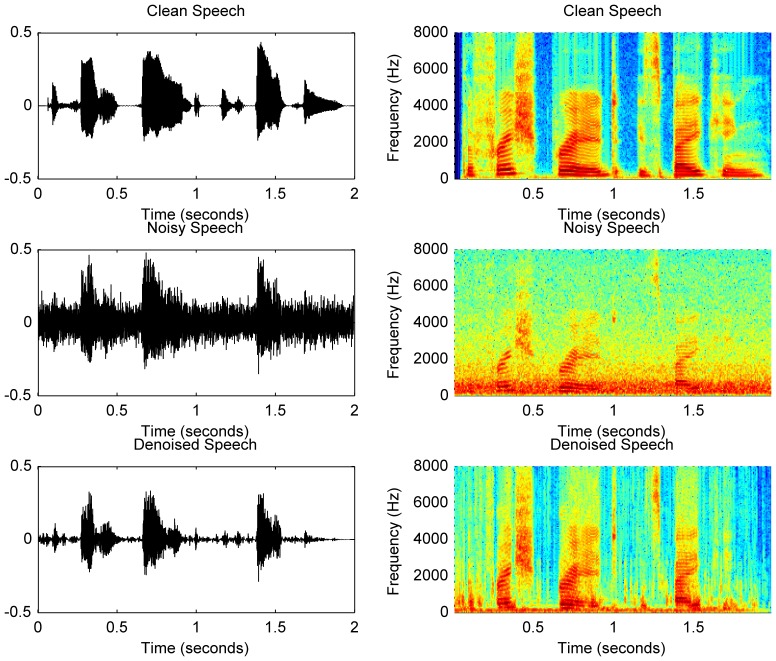
The time-domain waveforms and spectrograms of a sample speech signal. The sentence was “The fresh bread is baking.” The noisy version was corrupted with the speech weighted noise (SWN) at an SNR of 0 dB. The plots in the left are the time-domain waveforms, and those in the right are the spectrograms. The top row is the clean version. The middle row is the noisy version. The bottom row is the denoised version.

In **Figure 7**, the time-domain waveforms and the spectrograms of the clean, noisy and denoised versions of the sentence “The fresh bread is baking.” are provided as a visualization of the effectiveness of the DTDWT based denoising algorithm. It is evident in both time and frequency domains that the proposed algorithm is able to remove a large amount of noise while keeping the main features of the original signal unchanged. In particular, the spectrograms show that the proposed algorithm is very good at capturing the rapidly changing features of the original signal.

**Figure 8:**
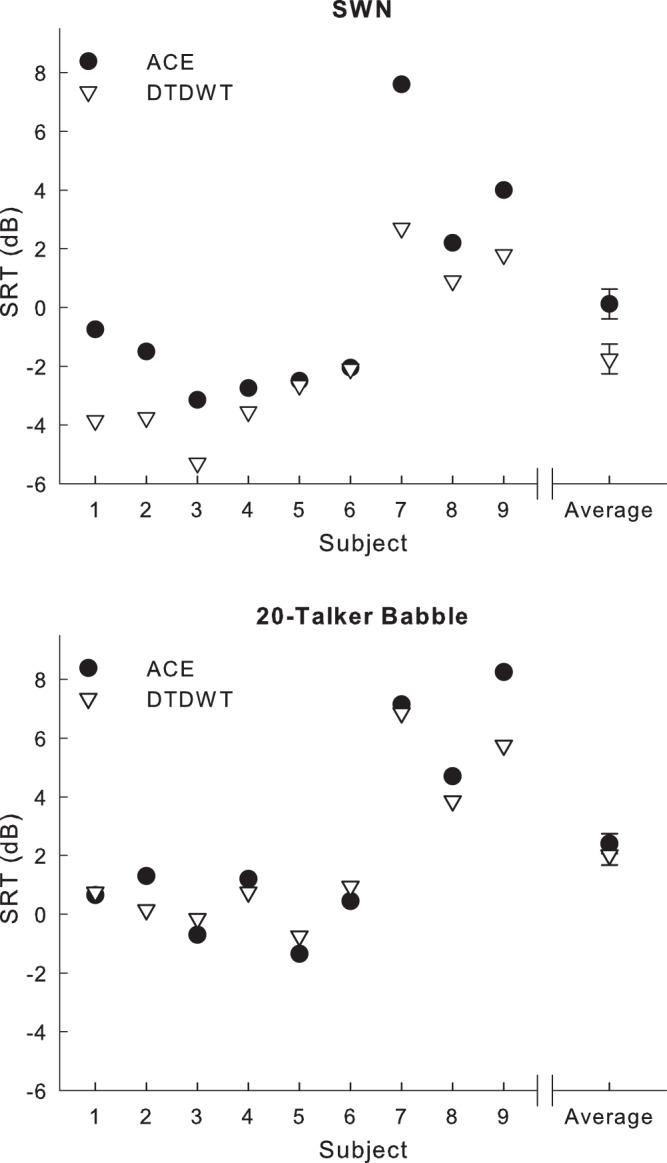
Subjects’ average speech reception threshold (SRT) results. The SRT (in dB) is defined as the SNR providing 50% morpheme intelligibility. The two noise conditions are the speech weighted noise (SWN) and 20-talker babble. Asterisks demonstrate a significant individual improvement at a 95% confidence level. Lower SRT scores show better performance.

Test results (**Figure 8**) show the speech performance results for nine subjects for the ACE condition and the DTDWT denoised condition. Mean scores in the SWN noise were 0.12 (ACE) and −1.76 (DTDWT) and in the 20-talker babble were 2.41 (ACE) and 2.02 (DTDWT). A two-way repeated measures analysis of variance (ANOVA) test was conducted on the main factors ‘program type’ and ‘noise type’. The two levels of ‘program type’ compared ACE and DTDWD and the two levels of ‘noise type’ compared SWN and 20-talker babble. The comparisons within the interaction factor ‘program type – noise type’, are particularly focussed on to determine any benefit of DTDWT over ACE in the different noise types. A significant main effect of ‘program type’ across both noise types (F [1], [8] = 11.35, p<0.01) was found. A significant main effect of ‘noise type’ across both program types was also found (F [1], [8] = 96.28, p<0.001).

A post hoc Newman-Keuls comparison showed that sentence perception in SWN was significantly improved by 1.9 dB for the DTDWT program compared to the ACE program (p<0.001). In this noise type, five of the nine subjects showed individual significant improvements above the 1.69 dB critical difference score for a 95% confidence level for our specific SRT test conditions [33]. No significant difference between programs was found in the 20-talker babble condition.

## Discussion

The clinical results in SWN conditions show that the wavelet-based noise reduction provides significant improvements in speech perception. A previous research study using a SNR-based noise reduction method showed a 2.1 dB SRT improvement in SWN [3]. The 1.9 dB SWN improvement in this study is more notable when considering the high performance (mean SRT +0.1 dB in ACE condition) of the subjects, who may receive smaller improvements from noise reduction [3]. Furthermore, no optimization of the shrinkage parameters for individual subjects was performed, such as increasing the aggressiveness of noise reduction [34] or increasing the temporal smoothing of noise reduction gain changes [4], which have shown to provide significant improvements for CI recipients. Therefore, it is expected that even better performance can be achieved when optimization of such parameters is performed.

The clinical results of the DTDWT in babble noise conditions showed no significant improvement over the ACE condition. One previous study has been able to show a significant speech understanding improvement in this noise type of 24 percentage points [2]. However, small or non-significant performance improvements have typically been found in babble environments with Fourier based noise reduction due to the high dynamics of the noise type [3], [4], [11]. A further reason for this result with the DTDWT is that the wavelet denoising algorithm was designed for SWN and its parameters were trained with the SWN corrupted speech data.

Simulation results with the NOIZEUS database indicate that the proposed algorithm performed better when compared to other conventional wavelet-based noise reduction algorithms under a variety of noise conditions. Further improvement would be achievable if separate sets of configuration parameters could be trained for a few typical noise environments and the system is able to select suitable configuration parameters according to some sound classification algorithm, such as the one proposed by Hu and Loizou [35].

This wavelet noise reduction algorithm was tested as a pre-processing system. Pre-processing testing has previously been useful in the initial assessment of noise reduction algorithms [8], but a “synergistic” implementation which does not require reconstruction into the time domain could be advantageous [7]. One advantage would be possible speech performance improvements since a synergistic implementation would not be effected by ‘musical noise’ from reconstruction [7]. Another advantage would be lower complexity and the ability to trial the noise reduction algorithm on a take home BTE device.

A number of research studies have suggested such a synergistic implementation where wavelet coefficients are used to determine stimulus output levels [19], [21]. However, clinical implementations currently require custom and adjustable filter bank boundaries due to patient individual preference for certain frequencies being mapped to electrodes. Using the denoised dual-tree wavelet coefficients directly to derive the stimulating signal with fully adjustable frequency bands remains a challenging task.

### Limitations of the Study, Open Questions, and Future Work

There are a number of limitations and open questions in this study. They are briefly discussed in the following to identify, where possible, ways to address them in future work.

- In its current form, the proposed algorithm will introduce a 0.5 second delay to the incoming signal. Therefore, it is not yet suitable for direct implementation in cochlear implants. This is an issue due to the algorithmic design in which many overlapping frames of signals are used to achieve the performance of the noise reduction. In addition, because the algorithm adaptively estimates the parameter for each frame of the signal, the length of the frame should be long enough to ensure the accuracy of the estimation. One possible way to address this problem is use intra-frame information for the estimation to explore the possibility of reducing the frame length and thus reducing the delay.
- In the clinical test, due to resource and time restrictions the proposed algorithm was only benchmarked against the state-of-the-art algorithm. The underlying assumption is that the simulation result and the STI index are good predictions of the clinical test performance of different wavelet-based noise reduction algorithm. Therefore, in this study different algorithms are first evaluated through simulation which is a cost-effective way to identify the candidate algorithm for clinical test. Subsequent to this, the candidate algorithm is then compared to the state-of-the-art in the clinical environment.
- A common limitation of all noise reduction algorithms is that they cause signal distortion as a byproduct of the noise removal process. An open question is then to try to quantify the effects of noise reduction and signal distortion in the particular application area of enhancing the speech intelligibility of persons with cochlear devices. Whilst this remains an important problem, it is out of the scope of this study. In this study, we have demonstrated the combined effects of both noise reduction and signal distortion. Clinical test results clearly show that the benefit of noise reduction outweighs the adverse effect of signal distortion. A future direction of this work is to use the denoised signal in the transform domain as the input to the cochlear device. This will serve to reduce signal distortion due to the modification of the signal in the transform domain, then transforming it back to the time domain.
- There are a few parameters in the proposed denoising algorithm. These parameters have been determined from simulations to maximize the overall STI for the dataset. Ideally, these parameters should be optimized for each patient. However, this would not be feasible in clinical practice.
- In this work, our study is focused on a particular type of noise: speech weighted noise (SWN). The algorithmic development and the training of the parameters are based on the SWN. Since different type of noise requires different algorithm to achieve the best result, the performance of the proposed algorithm is only optimum for SWN. A future direction of this study is thus to develop algorithms targeting other types of noise and develop an automated switching mechanism to select the algorithm to achieve the best results.

## Conclusions

In this study, a dual-tree wavelet-based noise reduction algorithm has been developed. Simulation experiments with SWN have demonstrated that the performance of proposed algorithm (based on the speech transmission index) is better than those of many existing wavelet-based algorithms. Clinical test results have also confirmed that the proposed algorithm resulted in significant speech performance outcomes in CI recipients. Further work in CI noise reduction with wavelet implementations should focus on using wavelet coefficients to drive stimulation levels and testing in a range of noise types.
