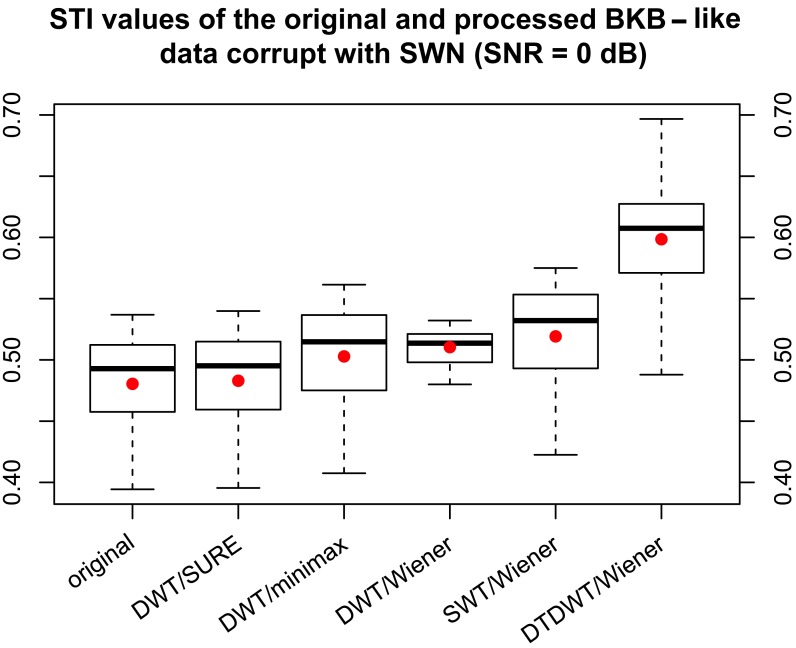# Correction: A Wavelet-Based Noise Reduction Algorithm and Its Clinical Evaluation in Cochlear Implants

**DOI:** 10.1371/annotation/13abca6d-18bb-451f-aa51-4f4f1d9c4dc9

**Published:** 2014-01-17

**Authors:** Hua Ye, Guang Deng, Stefan J. Mauger, Adam A. Hersbach, Pam W. Dawson, John M. Heasman

There was an error introduced during the preparation of this manuscript for publication. Figure 3 is incorrect. Please view the correct Figure 3 here: 

**Figure:**